# 10 Best resources for community engagement in implementation research

**DOI:** 10.1093/heapol/czx123

**Published:** 2017-10-30

**Authors:** Douglas Glandon, Ligia Paina, Olakunle Alonge, David H Peters, Sara Bennett

**Affiliations:** Johns Hopkins Bloomberg School of Public Health, Schiffer 615 North Wolfe Street, Baltimore, MD 21205, USA

**Keywords:** Implementation, community participation, research methods, stakeholders

## Abstract

Implementation research (IR) focuses on understanding how and why interventions produce their effects in a given context. This often requires engaging a broad array of stakeholders at multiple levels of the health system. Whereas a variety of tools and approaches exist to facilitate stakeholder engagement at the national or institutional level, there is a substantial gap in the IR literature about how best to do this at the local or community level. Similarly, although there is extensive guidance on community engagement within the context of clinical trials—for HIV/AIDS in particular—the same cannot be said for IR. We identified a total of 59 resources by using a combination of online searches of the peer-reviewed and grey literature, as well as crowd-sourcing through the Health Systems Global platform. The authors then completed two rounds of rating the resources to identify the ‘10 best’. The resources were rated based on considerations of their relevance to IR, existence of an underlying conceptual framework, comprehensiveness of guidance, ease of application, and evidence of successful application in low- or middle-income countries or relevant contexts. These 10 resources can help implementation researchers think strategically and practically about how best to engage community stakeholders to improve the quality, meaningfulness, and application of their results in order to improve health and health systems outcomes. Building on the substantial work that has already been done in the context of clinical trials, there is a need for clearer and more specific guidance on how to incorporate relevant and effective community engagement approaches into IR project planning and implementation.

Key Messages

- Meaningful, effective community engagement is often essential for implementation research. Yes, unlike the field of clinical trials research, little practical guidance exists on how implementation researchers can and/or should engage community stakeholders.
- The selected ‘10 best’ resources originate from both LMIC and high income country contexts and range from frameworks to standards to specific tools and ‘toolkits’. Conceptual foundations include appreciative inquiry, development theory, program theory evaluation, and others. We noted substantial cross-referencing between approaches but generally little discussion of how a researcher might determine if, how and when to apply them within a research project.
- The substantial variation in the types of resources available (purpose, scope, tools, targeted audience, resources required, etc.) calls for holistic, systematic consideration and specific guidance on how community engagement approaches can best be incorporated into each step of the implementation research cycle.

## What is implementation research and why is stakeholder engagement important?

Peters, Tran and Adam (2013) define implementation research as the ‘scientific study of the processes used in the implementation of initiatives as well as the contextual factors that affect these processes’. They further observe that implementation research (IR) should be collaborative, involving both implementers and decision-makers in the identification and design of a study as well as other phases of the research process (Peters, Tran and Adam 2013). It is increasingly recognized that blueprint approaches to implementation and scaling-up are not sufficient to acknowledge and manage the complexity in which we intervene (Peters, Tran and Adam 2013). On the contrary, flexible, adaptive, ‘learning by doing’ approaches that leverage partnerships with an array of health systems stakeholders are also necessary - not only to identify needed changes, but also to help ensure that those changes are translated into policy and practice. This arguably places stakeholder engagement approaches on par with research methods in terms of importance to the success of the research project. While there are multiple resources outlining key principles or general steps for stakeholder engagement, the highly contextual nature of the effort means that these are often challenging to implement in practice, particularly at the community level (Goodyear-Smith, Jackson, and Greenhalgh 2015). In this paper, we aim to identify resources to help those undertaking implementation research to engage different types of community stakeholders in the research process.

## What is community engagement?

‘Community’ may be understood as a group of people who live in the same local geographical area or who have some other non-spatial element of shared social identity, such as a similar trade or group membership (MacQueen *et al.* 2001; George *et al.* 2015). In this paper, we focus primarily on geographically-defined communities. Our definition however also encompasses organized entities that operate within a community such as local government, district health teams, or other community-based organizations, such as religious or civil society groups. ‘Community engagement’ in this context is the meaningful, respectful, and fit-for-purpose involvement of community members in one or more aspects of an IR project, and may include involvement during the identification of the study, to defining its purpose and design, to stages of implementation, interpretation, and use of results.

To operationalize the terms ‘meaningful and respectful’, we refer specifically to approaches that would be classified within the top three rungs of Arnstein’s ‘Ladder of Citizen Participation’—partnership, delegated power, citizens’ control—which outline the extent of power-sharing with individual people in a given initiative or political process (Arnstein 1969). In other words, perfunctory or tokenistic efforts to gain input from or disseminate results to community members would, for our purposes, not be considered community engagement. The term ‘fit for purpose’ encompasses elements of power sharing and inclusiveness. The former reflects our perspective that, while some degree of power sharing between the research team and communities is important, there are a variety of situations in which IR projects may be both technically and ethically sound even if they do not involve extensive empowerment of the community. This may be particularly relevant for IR projects that seek, for instance, to improve supply-side issues related to the technical quality or efficiency of an intermediate step in a health program or service, such as inventory management of medicinal products. The element of inclusiveness highlights the heterogeneity of members within a given geographic community and the need to explicitly identify and seek out diverse perspectives from relevant sub-groups. For instance, cultural sensitivities notwithstanding it would not be sufficient for an IR project on family planning access to work with a group consisting only of married women (i.e. to the exclusion of men, unmarried women, girls and boys)). In short, greater heterogeneity among individuals in the study area with respect to the IR topic and question(s) requires a more nuanced community engagement approach, which may also have time and resource implications.

## What is the role of community engagement in implementation research?

While the role and principles of community engagement in clinical trials research have been well documented (CTSA 2011; NBAC 2001; UNAIDS 2011), there is a lack of comparable guidance for IR. The above definition implies that the specific role of community engagement varies depending on the nature of the research question, the particular phase of the research cycle, and the preferred epistemology or paradigm of the research team. In terms of the research question, the key consideration is the extent to which local communities are directly involved in either providing or accessing the aspect of the intervention being studied. For instance, community engagement may be highly relevant to an IR project investigating barriers to scale-up of a voucher scheme promoting deliveries with a skilled birth attendant, but potentially less so for translational research in which the primary focus is a gap in specialist adherence to a clinical protocol for managing obstetric emergencies. That said, the strong IR emphasis on figuring out what actually works in ‘real-world’ settings (Peters *et al.* 2013) suggests that the experiences of local frontline workers and community-level beneficiaries are often critical for answering the research question.

Even for IR projects that involve a substantial community engagement component, the specific form of this engagement may vary substantially over the course of the project. Table 1, which is an adaptation of the ‘six steps of the implementation research cycle’ in the IR Toolkit developed by the World Health Organization through the Special Programme for Research and Training in Tropical Diseases (WHO/TDR 2014), provides some illustrative examples of the ways in which community engagement may add value to various phases of the IR process. In the problem identification phase, for instance, researchers may seek input from community members on the key problems or issues to be addressed by the research question or key stakeholders to include in the process. The implementation phase may include an intervention, and various data collection methods such as a household survey, focus groups or a participatory research component. Community stakeholders may also play a key role in the analysis and interpretation of findings (e.g. to provide local context to help explain an observed result) as well as in subsequent efforts to address identified issues or gaps. While this particular framework is focused on the research cycle, many of these phases overlap with implementation and policy cycles and could be adapted accordingly.

**Table 1.** Potential roles for community engagement by phase in the IR cycle

Phase in the IR cycle	Potential roles for community engagement
1. Problem identification	Input on key problems or issues to be addressed; understanding context, conceptualizing key issues; identifying key stakeholders to involve; conducting stakeholder mapping and analysis
2. Design and planning	Shaping key research aims, questions to meet local objectives; input into methodology, especially contextually appropriate approaches for data collection; review of research documents and tools (e.g., protocol, consent forms, instruments)
3. Implementation	Generating awareness and ownership of research project; potential involvement in an intervention being studied, pilot testing of instruments; participating as data collectors or respondents; formal partnership and collaboration with community groups
4. Analysis and interpretation	Interpreting findings; discussing implications; adding contextual depth and nuance to recommendations
5. Knowledge translation	Discussing implications of findings; issue prioritization, planning and implementation of follow-up action; tailoring evidence to enhance community voice
6. Iteration and adaptation	Establishing ongoing community participatory M&E, social accountability mechanisms to increase transparency of key service delivery outcomes

Regardless of the step in the IR cycle, the potential role of community engagement is also influenced by the epistemological perspective or worldview of the research team. For those who subscribe to a constructivist or pragmatic paradigm, decisions about specific approaches to community engagement are likely to figure prominently in methodological discussions since results are understood to be dependent (at least in part) upon the experiences and perceptions of the respondents. In a participatory paradigm, community engagement may take on even more of a central role, as the motivation to improve social conditions or reduce marginalization may supersede generalizability or transferability of results. On the other hand, some post-positivists may be primarily interested in obtaining community input for the purposes of identifying appropriate instrumental variables to strengthen causal inference or increasing survey response rates to generate adequate sample size. Of note, this latter interpretation does not meet our definition of ‘community engagement’, as it implies a lower level of participation—a transactional exchange rather than an effort to facilitate power-sharing. This also highlights the fact that community engagement may be a more natural fit for some research paradigms than others.

## Process for selecting community engagement resources

We identified a total of 59 resources to review through a combination of: a) crowd-sourcing through the health systems working group ‘SHAPES’ (Social science approaches for research and engagement in health policy and systems) of Health Systems Global (HSG 2016), an international membership organization promoting health systems research; b) depersonalized Google and Google Scholar searches, and; c) snow-ball searches of the identified peer-reviewed literature using keywords in PubMed, Medline, Scopus, Embase, Web of Science, PsychInfo, and EconLit (Supplementary Annex). To review resources, we prepared a matrix of key attributes/criteria for each resource, including the creator, creation date, purpose, country context (high income, low and middle income, or both), theoretical foundation or framework, targeted users and stakeholders, resource requirements, targeted stakeholders, stage of implementation, level of engagement, and evidence of application. The authors then completed two rounds of rating the resources. Ratings in the first round were based on the relevance of the resource to community engagement in IR, as defined above (based on stated purpose, targeted users, and potential applicability to a research project for at least one of the six phases listed in Table 1), which narrowed the list of resources from 59 to 23 (Supplementary Annex). All ratings were discussed by three members of the research team to generate a consensus rating for each resource. The 23 resources that were reviewed in-depth were diverse in their origins, purpose, format, and audience. Many came from the broader development literature, but also from evaluation theory, participatory action research and systems thinking. Purposes ranged from identifying local research priorities to community empowerment, with a broad spectrum in between. A mixture of frameworks, guidelines, techniques, and ‘toolkits’ were targeted at researchers, practitioners, trainers, community groups, or some combination thereof. We further excluded 8 out of the 23 identified resources due to lack of published literature regarding their use. One resource—the Implementation Research Toolkit—was retained despite limited evidence of application because of its explicit focus on implementing research projects. In the second round, the remaining 15 resources were rated again to identify the ‘10 best’ based on the availability and robustness of the conceptual framework, the comprehensiveness of guidance provided, apparent ease of application, and evidence of successful application in low- or middle-income countries or relevant contexts. In addition to these individual criteria, we sought to ensure a diversity of resources encompassing different stages of the research cycle or might be useful for different types of research projects.

## 10 Best resources

The final 10 resources were not ranked in any particular order but rather selected based on their collective and complementary value for IR teams interested in community engagement. By ‘IR team’, we mean the group of people collaborating to conduct a particular IR project, including researchers, coordinators and administrative staff, key counterparts in partner organizations, community representatives, or others. Since only one resource explicitly referenced implementation research—the Implementation Research Toolkit (WHO/TDR 2014)—our focus was on the potential contribution of a given resource to *at least one stage* of the IR process (Table 1). As mentioned above, our interpretation of community engagement includes a meaningful degree of power-sharing between the IR team and the communities involved in or affected by the research. Whether or not this actually happens depends on the research team rather than on any given resource but we selected resources in which this was either an explicit aim or a logical application. There are also a few instances in which other resources within the set of 59 serve a similar purpose to the ‘10 best’, which we have noted in the text below. A list of the ‘10 best’ resources along with illustrative applications and a brief summary of country case studies or examples is outlined in Table 2.

**Table 2.** 10 Best resources for community engagement with illustrative applications

#	Resource	Format	Illustrative Applications	Country/case example
1	Principles of Community Engagement	Standards/ Guidelines	Consider a range of conceptual, ethical and practical issues relevant to community engagement in an IR project.	US (Lake County, Chicago): study team formed community advisory committee to create a shared mission statement and adapted study design to meet community needs. (CTSA 2011)
2	Participatory Poverty Assessment	Guide with case studies	Determine how the study will ensure adequate and equitable representation from underserved intervention beneficiaries.	Uganda: a Participatory Poverty Assessment process was undertaken to incorporate voices of the poor into Uganda’s Poverty Eradication Action Plan, with a 3-yr process to link the findings to central and district-level policy making. (Norton *et al*. 2001)
3	Systems Concepts in Action	Primer with case examples	Apply systems thinking methods and tools to understand and analyze complex systems dynamics and relationships associated with the intervention.	East Tyrol, Austria: a Strategic Area Assessment was used to guide a range of stakeholders in rapidly generating a holistic picture of development potentials of the rural, mountainous region and was embedded within a participatory strategy building process. (Williams and Hummelbrunner 2011)
4	Implemen-tation Research Toolkit	Facilitator Guide & Participant Manual	Train local research team in fundamentals of IR and specific methodological aspects of a particular study to be conducted. Users may tailor the toolkit to add content on community engagement approaches.	No specific case studies, examples or scenarios documented.
5	Participatory Impact Pathways Analysis (PIPA)	Guide/ Manual	Engage a variety of stakeholders, including at the community level, to articulate a shared theory of change and draw actor network maps, which will form the basis of the IR questions and metrics.	Vung Tau, Viet Nam: A PIPA workshop with key public, private and development partners resulted in a shared, theory-based five-year vision for scaling up successful pilot interventions to reduce postharvest losses and a design for a multi-stakeholder learning/M&E platform. (Schütz *et al*. 2009)
6	Engagement Toolkit, v.4	Inventory with user notes	Review a broad array of specific approaches and techniques for community engagement and select or adapt those most suitable for a given IR project.	‘Santa Rosa’, country not specified: A project team including local health facility staff randomly selects health clinic users to brainstorm problems and then anonymously vote based on frequency, importance and feasibility of solving the problem to prioritize issues for the project team to address (MSH, UNICEF 1998)
7	Most Significant Change (MSC)	Guide/ Manual	Gather qualitative data on community perceptions of the most important intended and unintended outcomes of an intervention; learn about stakeholder values/priorities.	Victoria, Australia: a collaborative dairy extension program working with farmers to improve farm productivity used MSC to understand impact of program on farmers’ lives across several ‘domains of change’. Stories were discussed as part of existing meetings, eventually highlighting very different perceptions of important outcomes by different stakeholders. (Davies and Dart 2005)
8	Social mapping; Net-Map	Guide/ Manual	Describe, analyze and monitor the influence of community actors and social networks on the implementation and outcomes of a particular intervention being studied.	Katsina, Nigeria: Net-Map interviews conducted with state government staff and stakeholders to explore the disconnect between newborn survival policies and actual funding and implementation. Results highlighted a divide between health sector actors making the plans and non-health actors allocating funds, resulting in actor-specific advocacy strategies. (Schiffer **et al*.* 2012)
9	Participatory Statistics	Primer with case studies	Use participatory approaches while maintaining statistical rigor in study design & analysis to achieve local ownership as well as broader policy relevance.	Mombasa, Kenya and Estelí, Nicaragua: a participatory climate change adaptation appraisal was applied to gather both quantitative and qualitative data on urban resident perceptions of assets, vulnerabilities and priorities to inform local policy debates on climate change adaptation efforts in urban centers. (Holland 2013)
10	Community Score Card (CSC)	Guide/ Manual	Facilitate community-based participatory monitoring of local service providers to enhance transparency and accountability.	Bamyan, Takhar and Nangarhar provinces, Afghanistan: the CSC was implemented as a social accountability mechanism to engage community members in monitoring service delivery, which resulted in participatory problem solving, increased trust in providers, and enhanced community solidarity. (Edward *et al*. 2015)

### Principles of Community Engagement, second edition

As defined by the Clinical and Translational Science Awards Consortium (CTSA) Community Engagement Key Function Committee Task Force of the National Institutes of Health (CTSA 2011), community engagement is ‘the process of working collaboratively with and through groups of people affiliated by geographic proximity, special interest, or similar situations to address issues affecting the well-being of those people’. This is derived from the view that health is largely socially determined and guided by values of ‘fairness, justice, empowerment, participation, and self-determination’ (CTSA 2011). The document briefly summarizes theoretical foundations from the literature and outlines nine principles, with practical examples of each, to guide researchers and practitioners in their own community engagement efforts (CTSA 2011).

The principles presented are clear, insightful, and applicable across a wide variety of settings, even if the authors and examples are primarily US-based. This document may be particularly useful for IR teams that are new to community engagement approaches, as it outlines a range of conceptual, ethical and practical issues to consider during research planning and implementation. These include, for instance, learning about community culture and context and perceptions of those initiating the engagement, recognition and acceptance of the importance of collective self-determination, and others. This is likely to be most useful in phases 1–3 of the IR cycle (Table 1).

### A rough guide to participatory poverty assessment

In *A Rough Guide to PPAs*, Norton *et al.* (2001) define the Participatory Poverty Assessment as ‘an instrument for including poor people’s views in the analysis of poverty and the formulation of strategies to reduce it through public policy’. The authors view poverty as a multi-dimensional construct with strong contextual dependence (Norton *et al.* 2001). Implicit within this perspective is the need for participatory methods to understand community members’ own perceptions of well-being and poverty. These findings in turn are used to inform national policies and strategies for poverty reduction (Norton *et al.* 2001).

While the explicit poverty focus of the participatory poverty assessment (PPA) is not inherent to IR, the emphasis on seeking input from the least well off in society to better understand their needs and preferences is likely to be highly relevant for many IR projects. The PPA conceptual approach and discussion may help IR teams reflect on how/where their own project may contribute to a broader national poverty reduction agenda. Implementing or adapting specific PPA methods may help ensure adequate and equitable representation from underserved intervention beneficiaries. This resource may be most useful in the first three phases if the IR cycle (Table 1).

### Systems Concepts in Action: A Practitioner’s Toolkit

This book by Williams and Hummelbrunner (2011) provides an overview of systems thinking and an introduction to a variety of specific systems thinking methods, including background information, case examples and ‘how to’ guidance. The aim is to help researchers and practitioners apply these methods and tools in their own context to understand and analyse complex systems dynamics and relationships associated with a given intervention. The book includes nineteen separate methods, based on the criteria that they are practical, tested, wide-ranging and multidisciplinary. Recommended further readings are also provided for each method.

The fact that IR must take into account the local context of interventions means that complexity is often an inherent part of the research. Systems Context in Action may serve as a useful starting point to help IR teams reflect on the level of complexity of the service or intervention under investigation. The introduction to systems thinking may help IR teams identify critical community stakeholders and perspectives to incorporate in the project and the showcased methods offer a menu of approaches that may be applicable to a variety of implementation research projects.

### Implementation Research Toolkit

The Implementation Research Toolkit (WHO/TDR 2014) is intended to serve as a training resource for IR teams, designed in particular to support individuals and institutions in low and middle income countries. The content, which is intended to be delivered in a workshop format, is organized according to the key steps involved in conducting IR and comes as a packaged set of a Facilitator’s Guide, Participant’s Manual and an accompanying slide deck.

Given that IR teams are often interdisciplinary and may include members with varying understanding of community engagement, some form of training session may be useful to make sure everyone has a clear and common understanding of the purpose and approach(es). The IR toolkit prompts the team to identify all relevant stakeholders and continuously emphasizes the importance of appropriate stakeholder engagement at each step in the research process. This provides a convenient foundation for users to tailor the training sessions to incorporate additional material or guidance related to community engagement.

### Participatory Impact Pathways Analysis

As described by Douthwaite *et al.* (2008), Participatory Impact Pathways Analysis (PIPA) is a ‘practical planning, monitoring and evaluation approach developed for use with complex projects in the water and food sectors’, but is equally relevant to the health sector. One central component is a facilitated, participatory workshop in which key stakeholders are convened to collaboratively articulate their understanding of a particular issue, the expected causal linkages in the ‘impact pathways’ of one or more interventions to address that issue, and a set of network maps to show important relationships between key actors or stakeholders. These workshop products then become the basis for identifying appropriate progress milestones and targets to track over time and update as needed.

Clarification of the expected causal pathway or logical framework of a given intervention is often a key component of IR projects. The PIPA materials can help IR teams facilitate this process using a participatory approach to incorporate the perspectives of key stakeholders, including at the community level. The network mapping component incorporates a complementary methodology, Net-Map, which is also included in this ‘10 best’ list and described further below. Of note, the PIPA approach is very similar to Outcome Mapping (Earl *et al.* 2001). Our collective experience with the use of PIPA found it a flexible tool that effectively enabled stakeholders to define their vision for project success and to monitor progress against this vision (Ekirapa-Kiracho *et al.* forthcoming). The design of the PIPA workshop can be adapted (in terms of length, breadth, and participation) to fit the needs and resource availability of the teams using it (STEPS 2017).

### Social Mapping/Net-Map

Net-Map (Schiffer *et al.* 2012) is a participatory approach to generating social network maps illustrating key actors involved in a particular system or issue, the relationships between them, their relative influence, and their respective goals. Network maps can be developed with participants individually or in groups and may have a variety of applications, ranging from developing a stakeholder management approach for an intervention to generating a shared understanding of a project, and so on.

As noted above in the commentary about PIPA, this approach can be used during the planning stages of an IR project to map out the current network of actors and their influence in a given intervention or issue. Using this methodology to periodically update the network map with relevant stakeholders may allow IR teams to incorporate network-based outcomes or indicators into the project, including at the community level. Manually created network maps may also be digitally recorded and analysed using social network analysis software.

### The Engagement Toolkit, Version 4

The Engagement Toolkit (DEPI 2014) is a collection of 68 specific tools and techniques for community engagement, each including a brief overview and summary of the associated objectives, uses, strengths and weaknesses, methods notes, and logistical details (e.g. cost, skill level, time required, audience size, etc.). The techniques range from traditional public meetings and printed materials to more recent approaches like computer-assisted systems modelling and ‘electronic democracy’. Users are intended to select the tool or tools that best fit their community context and adapt them as needed.

As described above, the role of community engagement in IR may vary substantially depending on the context. This toolkit provides a quick reference for IR teams to review a broad array of specific approaches and techniques for community engagement and then select or adapt those that are most suitable for a given IR project. Users may seek out further information and guidance on techniques of interest from other sources.

### Most Significant Change

The Most Significant Change (MSC) approach was designed by Davies and Dart (2005) as a form of participatory monitoring and evaluation to help address the limitations of focusing solely on logical framework-derived indicators, especially when working with complex programs and environments. This approach involves the reporting of ‘stories of change’ by program participants, beneficiaries and field staff, followed by multiple stages of group review in which designated stakeholders (e.g. program staff, funders) identify the stories considered most significant and then follow up with field visits and further investigation to verify and quantify those changes.

A key strength of this approach is that it does not constrain evaluation to only planned or desired outcomes; it can also capture unintended consequences of an intervention. If applied at the community level, MSC can generate open-ended dialogue about community perceptions of the most important (intended and unintended) outcomes of an intervention. In addition to generating qualitative data about an IR project at the local level, this approach can provide insights into community values and priorities and also inform the interpretation of the findings of IR projects. Of note, this approach is very similar to Outcome Harvesting (Wilson-Grau and Britt 2012).

### Participatory Statistics

The term ‘participatory statistics’ refers to a broad range of methods that follow a participatory approach to data collection, following the paradigm of participatory research (Holland 2013). The core concept is the idea of a ‘win–win’ scenario in which the monitoring, evaluation, and assessment of international development projects are empowering and transformative for the local people involved while also incorporating statistical principles to produce representative, generalizable results. An introductory chapter explaining the concept is followed by a series of country case studies using various methods.

This resource may be particularly useful for IR teams or projects with a primarily quantitative focus but also an interest or need to incorporate meaningful community engagement. While this resource does not provide a ‘how to’ guide for any particular resource, IR teams may find it useful to read through the case studies for ideas about approaches that may be most relevant for their own project.

### Community Score Card 

In addition to facilitating community-level participatory monitoring of a local programs and services, the Community Score Card (CARE 2013) is intended to serve as a means of enhancing citizen voice and increasing transparency, accountability and responsiveness of service providers. The process involves service users and providers coming together in a facilitated discussion to identify service delivery issues and develop a shared understanding of how best to address those issues.

The Community Score Card (CSC) is an example of how IR teams may approach community-level monitoring and evaluation not only as a means of gathering data on service delivery outcomes but also as an intervention in itself to influence those outcomes. The strong emphasis on local voice and empowerment exemplifies the principle of power-sharing highlighted in the Participatory Poverty Assessment and the Principles of Community Engagement. With systematic sampling and scoring, the CSC may also help IR teams achieve the ‘win-win’ scenario sought by the Participatory Statistics approach.

## Conclusion

Despite the frequent mention of the importance of stakeholder engagement in IR, there is limited practical guidance for how best to engage communities in IR, including who, how, when to engage, for how long, in what ways, etc. We hope that these 10 resources will help implementation researchers think strategically and pragmatically about when and how best to engage community stakeholders to improve the quality, meaningfulness, and application of their results to improve health and health systems outcomes in the study locations, as well as to stimulate discussion about the appropriate role of community engagement approaches as an explicit methodological consideration in implementation research projects.

We noted substantial overlap and cross-referencing of approaches but very little discussion of how a researcher might determine if, how, and when to apply them within a research project. Frequently we struggled to classify the resource according to the stage of the implementation research process that it might be used. There is a general gap in empirical documentation of these approaches. Going forward, it would be beneficial for teams conducting research involving community engagement—including IR teams but also those doing community-based participatory research, participatory action research, etc.—if the producers of such resources are clearer about the purpose of their tool/approach and document their applications with reflection on what worked (or did not work) and why, as well as suggestions for how to overcome challenges associated with engaging communities. Additionally, there is a need for further assessment and understanding of the competencies needed for IR teams to effectively employ various community engagement approaches, including many of the approaches listed here. This would not only help individual IR teams plan for particular projects but would also be a valuable contribution to the curricula for various IR teaching and training programs to ensure that new researchers are better equipped to deliberately and thoughtfully engage with communities affected by their work in ways that are respectful and empowering.

These 10 best resources call attention to the importance of flexible, nuanced strategies and clear, practical guidance for research teams seeking meaningful community engagement. They also provide a foundation for further inquiry on how and when to engage communities, including discussion of how the IR community can promote further learning and capacity development in this area.

## 10 Best resources

CARE Malawi. 2013. *The Community Score Card (CSC): A generic guide for implementing CARE’s CSC process to improve quality of services*. Atlanta, Georgia: Cooperative for Assistance and Relief Everywhere, Inc. http://www.care.org/sites/default/files/documents/FP-2013-CARE_CommunityScoreCardToolkit.pdf

CTSA Community Engagement Key Function Committee Task Force. 2011. *Principles of Community Engagement, Second Edition.* Bethesda, Maryland: National Institutes of Health. https://www.atsdr.cdc.gov/communityengagement/pdf/PCE_Report_508_FINAL.pdf

Davies R, Dart J. 2005. *The ‘Most Significant Change’ Technique: A guide to its use*. www.mande.co.uk/docs/MSCGuide.htm

Department of Environment and Primary Industries (DEPI), State of Victoria, Australia. 2014. The Engagement Toolkit, Version 4 - Effective Engagement: building relationships with community and other stakeholders. Melbourne, Australia: DEPI. http://www.dse.vic.gov.au/__data/assets/pdf_file/0003/105825/Effective_Engagement_4_-_Book_3_v3-01.pdf

Douthwaite B, Alvarez S, Tehelen K, Cordoba D, Thiele G, Mackay R. 2008. Participatory impact pathway analysis: A practical method for project planning and evaluation. In: *Fighting poverty through sustainable water use*. Proceedings of the CGIAR Challenge Program on Water and Food 2nd International Forum on Water and Food, Vol. 4, Addis Ababa, Ethiopia, 10-14 November 2008, eds. R.S. Bayot and E. Humphreys; 31. Colombo, Sri Lanka: CGIAR Challenge Program on Water and Food. http://pipamethodology.pbworks.com/w/page/70283575/Home%20Page

Holland J. 2013. *Who Counts? The Power of Participatory Statistics*. Institute of Development Studies. Warwickshire, UK: Practical Action Publishing. http://www.ids.ac.uk/publication/who-counts-the-power-of-participatory-statistics

Norton A, Bird B, Brock K, Kakande M, Turk C. 2001. *A rough guide to PPAs: Participatory Poverty Assessment – an introduction to theory and practice*. London: Overseas Development Institute. https://www.odi.org/publications/1747-rough-guide-ppas-participatory-poverty-assessment-introduction-theory-practice

Schiffer E. 2007. *Net-Map toolbox: influence mapping of social networks*. International Food Policy Research Institute. Paper presented at the Sunbelt Conference of the International Network of Social Network Analysis, Corfu, Greece, 01-06 May 2007. https://netmap.files.wordpress.com/2008/06/net-map-manual-long1.pdf

WHO/TDR. 2014. *Implementation research toolkit*. Geneva, Switzerland: World Health Organization on behalf of the Special Programme for Research and Training in Tropical Diseases. http://www.who.int/tdr/publications/year/2014/9789241506960_workbook_eng.pdf

Williams B, Hummelbrunner R. (2011). *Systems Concepts in Action: A Practitioner’s Toolkit*. Stanford: Stanford University Press. http://www.sup.org/books/title/?id=18331

## Ethical approval

Not applicable as this is a secondary review of existing literature; not human subjects research.

## Supplementary data

Supplementary data are available at *HEAPOL* online.

## Supplementary Material

Supplementary Data
