# Supplementary material for: 10 Best resources for community engagement in implementation research
Source: Health Policy Plan. 2017 Oct 30;32(10):1457–65. doi: 10.1093/heapol/czx123 (PMC5886100; doi:10.1093/heapol/czx123)
Supplement: Supplementary Data [file supplementary_file_revised_czx123.docx]

**Web Annex: Community Engagement Resources Reviewed**

In the following table, the resources identified as “10 best” are bolded. All resources that were selected into the second round of review are highlighted in gray. Key words for the search included combinations of the following: approach, community, empowerment, engagement, framework, guidance, guide, guidelines, implementation, involvement, participation, participatory, research, resource, stakeholder, technique, tool, toolkit, voice.

| **Tool/Resource name** | **Creator/Author** | **Creation Date** |
| --- | --- | --- |
| *A tool to analyze the transferability of health promotion interventions* | *Cambon L, Minary L, Ridde V, Alla F* | *2013* |
| Active Community Engagement (ACE) Continuum | Russell R, Igras S, Johri N, Kuoh H, Pavin M, Wickstrom J | 2008 |
| CIHR Guidelines for Health Research Involving Aboriginal People | Canadian Institutes of Health Research (CIHR) | 2007 |
| *Collaboration Multiplier* | *Prevention Institute* | *2011* |
| Community development as organizational learning | Dyck B, Buckland J, Harder H, Wiens D | 2000 |
| Community engagement: improving health and wellbeing and reducing health inequalities | National Institute for Health and Care Excellence (NICE) | 2016 |
| Community Engagement How-To Guide | Scottish Government | 2014 |
| Community engagement in research: frameworks for education and peer review | Ahmed SM, Palermo AS | 2010 |
| Community Engagement Toolkit | Social Planning and Research Council of British Columbia | 2013 |
| Community Health Worker Evaluation Toolkit | University of Arizona Rural Health Office and College of Public Health | 1998 |
| *Community Scoping* | *Research Consortium on Educational Outcomes and Poverty (RECOUP)* | *2008* |
| *Complexity-aware monitoring* | *Britt H* | *2016* |
| Consent and community engagement in diverse research contexts: reviewing and developing research and practice | Molyneux S, Bull S, Cheah PY, Lwin KM, Marsh V, Parker M, Theobald S | 2013 |
| *Development of a tool for planning and evaluating action research on health in Burkina Faso* | *Aka BRS, Queuille L, Pilabre F, Zidwemba N, Ridde V* | *2015* |
| Effective Engagement: A Guide to Principles and Practices | Effective Interventions Unit of Scottish Government | 2002 |
| Engagement for Impact: Community Stakeholder Engagement (CSE) Monitoring & Evaluation | Oliff M, Babigumira K | 2015 |
| *Equal Access Participatory M&E Toolkit* | *Lennie J, Tacchi J, Koirala B, Wilmore M, Skuse A* | *2011* |
| Ethical conduct for research involving humans | Canadian Institutes of Health Research (CIHR) | 2014 |
| Evaluating community engagement in global health: the need for metrics | MacQueen KM, Bhan A, Frolich J, Holzer J, Sugarman J | 2015 |
| Expanding the prevention armamentarium portfolio: A framework for promoting HIV Conversant Communities within a complex, adaptive epidemiological landscape | Burman CJ, Aphane M, Mtapuri O, Delobelle P | 2015 |
| Exploring Challenges, Progress, and New Models for Engaging the Public in the Clinical Research Enterprise | Aungst J, Haas A, Ommaya A, Green LW | 2003 |
| Grand Challenges in Global Health: Community Engagement in Research in Developing Countries | Tindana PO, Singh JA, Tracy CS, Upshur REG, Daar AS, Singer PA, Frohlich J, Lavery JV | 2007 |
| Guide to Researcher and Knowledge-User Collaboration in Health Research | Parry D, Salsberg J, Macaulay AC | 2015 |
| Handbook on Community-Led Total Sanitation (CLTS) | Kar K, Chambers R | 2008 |
| IAP2 Spectrum of Public Participation | International Association for Public Participation (IAP2) | 2014 |
| **Implementation Research Toolkit** | **Special Programme for Research and Training in Tropical Diseases (TDR), World Health Organization** | **2014** |
| Inclusive rigour for complexities (participatory methodologies) | Chambers R | 2015 |
| *Interactive community mapping: Between empowerment and effectiveness* | *Shkabatur J* | *2014* |
| Knowledge Sharing Toolkit | CGIAR, FAO, KM4Dev Community, UNICEF, UNDP | 2016 |
| Measuring empowerment | Narayan D | 2005 |
| **Most Significant Change (MSC)** | **Davies R, Dart J** | **2005** |
| **Net-Map toolbox: influence mapping of social networks** | **Schiffer E** | **2008** |
| *Origins and practice of Participatory Rural Appraisal (PRA)* | *Chambers R* | *1994* |
| Outcome harvesting | Wilson-Grau R, Britt H | 2012 |
| *Outcome mapping* | *Earl S, Carden F, Smutylo T* | *2001* |
| *Participation Toolkit* | *Hunt S, Spreckley F* | *2005* |
| *Participatory Action Research* | *Loewenson R, Laurell AC, Hogstedt C, D’Ambruoso L, Shroff Z* | *2014* |
| **Participatory Impact Pathways Analysis (PIPA)** | **Douthwaite B, Alvarez S, Tehelen K, Cordoba D, Thiele G, Mackay R** | **2008** |
| **Participatory Poverty Assessment** | **Norton A, Bird B, Brock K, Kakande M, Turk C** | **2001** |
| **Participatory Statistics** | **Holland J** | **2013** |
| Participatory Workshops: A Sourcebook of 21 Sets of Ideas and Activities | Chambers R | 2002 |
| **Principles of Community Engagement, 2^nd^ edition** | **CTSA, NIH** | **2011** |
| Process and outcome constructs for evaluating CBPR projects | Sandoval JA et al. | 2012 |
| *Rapid rural appraisal: rationale and repertoire* | *Chambers R.* | *1981* |
| Recommendations for community engagement in HIV/AIDS research | Community Partners, NIH | 2014 |
| Rich Pictures (in Soft Systems Methodology in Action) | Checkland P, Scholes J. | 1999 |
| SALT approach | The Constellation | 2016 |
| SARAR Process (in Tools for Community Participation) | Srinivasan L | 1993 |
| Self-Evaluation for Effective Decision-making (SEED) System for Communities to Adapt Learning and Expand (SCALE) | Taylor-Ide D, Taylor CE | 2002 |
| Stakeholder assessment (in Enhancing Organizational Performance: A Toolbox for Self-Assessment) | Lusthaus C, Adrien MH, Anderson G, Carden F | 1999 |
| Stakeholder engagement toolkit for HIV prevention trials | MacQueen KM, Harlan SV, Slevin KW, Hannah S, Bass E, Moffett J | 2012 |
| **Systems concepts in action** | **Williams B, Hummelbrunner R** | **2010** |
| Taking tissue seriously means taking communities seriously | Upshur REG et al. | 2007 |
| **The Community Score Card** | **CARE Malawi** | **2013** |
| **The Engagement Toolkit, Version 4** | **Department of Environment and Primary Industries (DEPI), State of Victoria, Australia** | **2014** |
| Towards a framework for community engagement in global health research | Lavery JV et al. | 2010 |
| *Useful tools for engaging young people in participatory evaluation* | *Gawler M* | *2005* |
| Voice and Accountability Tool: Engagement Framework | Catholic Aid Agency for England and Wales (CAFOD) | 2008 |
| What makes clinical research in developing countries ethical? Benchmarks of ethical research | Emanuel et al. | 2004 |
